# On a noteworthy habitat type in the savannahs of Central Cuba and a remarkable new species of Elytraria (Acanthaceae)

**DOI:** 10.3897/phytokeys.177.64764

**Published:** 2021-05-13

**Authors:** Werner Greuter, Rankin Rodríguez

**Affiliations:** 1 Botanischer Grten und Botanisches Museum Berlin, FU Berlin, Germany Botanischer Grten und Botanisches Museum Berlin Berlin Germany; 2 Jardín Botánico Nacional, Universidad de La Habana, La Habana, Cuba Universidad de La Habana La Habana Cuba

**Keywords:** *
Acanthaceae
*, Cuba, *
Elytraria
*, “mocarrero” soil, myrmecophyly, new species, “perdigón”, vegetative propagation, Villa Clara Province

## Abstract

A peculiar habitat type found in the savannahs of Central Cuba, Villa Clara Province and characterised by the presence of a surface gravel layer of “perdigones”, an assemblage of small ferralitic concretions, upon the “mocarrero” soil prevailing in the area, is described. On sterile gravel patches, only one species grows: *Elytraria serpens*, a new species described and named here. It is noteworthy for possessing long and wide creeping, stoloniform subterranean peduncles with apical gemmae developing into rooting leaf rosettes enabling vegetative propagation. The new species is close to *E. shaferi* and considered to derive from the latter by adaptive evolution, enabling it to survive in its hostile habitat, sheltered from the competition of other plant species. Small soil insects, for example, ants, are believed to act as pollinators.

## Abstract (es)

Se describe un hábitat particular encontrado en la provincia Villa Clara, caracterizado por la presencia de una capa superficial de perdigones (pequeñas concreciones ferralíticas) que recubre el suelo mocarrero de aquella área. En los parches estériles con grava solo crece *Elytraria serpens*, nueva especie aquí descrita, notable por tener pedúnculos subterráneos largamente rastreros y reproducirse vegetativamente por yemas apicales que forman rosetas satélites que luego se enraízan. La nueva especie es afín a *E. shaferi* y se considera derivada de esta por evolución adaptativa, permitiéndole la colonización de un hábitat desfavorable, abrigada de la competencia de otras especies vegetales. Se opina probable la polinización por pequeños insectos terrícolas, p. ej. hormigas.

## Introduction

Amongst many interesting Cuban localities that the authors visited in March 2019, under the expert guidance of botanists of the Botanical Garden, Universidad Central “Marta Abreu” of Las Villas, the one that we want to present here stands out, not only for hosting a species new to science that we are going to describe hereunder, but also by the particular aspect of its soil. Villa Clara botanists were already familiar with that very locality because of the presence in it of a small population of *Paspalum edmondii* León, a rare endemic species, listed as Critically Endangered (González Torres et al. 2016). This site is located in the Corralillo Municipality, at 22°50'55" latitude North and 80°28'22" longitude West, at an elevation of ca. 65 m a.s.l., between the Las Cañas and Clarita Rivers (or brooks). According to Borhidi’s (1991: 344) phytogeographic typology, it situated within the “Sagüense District” that encompasses the hills and plains of the northern coastal strip of Las Villas.

The soils of the area are of the “mocarrero” type, which, according to Bennett and Allison (1928: 67), is characterised by the presence of a 10–25 cm thick ferralitic rock layer (“ironstone”) at a depth of 25 to 100 cm, a layer that inhibits drainage and causes the lands, very arid in the dry season, to become swampy in the rainy season. This, added to the general soil oligotrophy, results in the prevailing of savannah plant formations (Bennett and Allison 1928; Borhidi 1991: 149). Borhidi (1991: 162) believes that the savannahs have replaced an original scrub vegetation, following an alternation of fire and grazing events.

**Figure 1.**
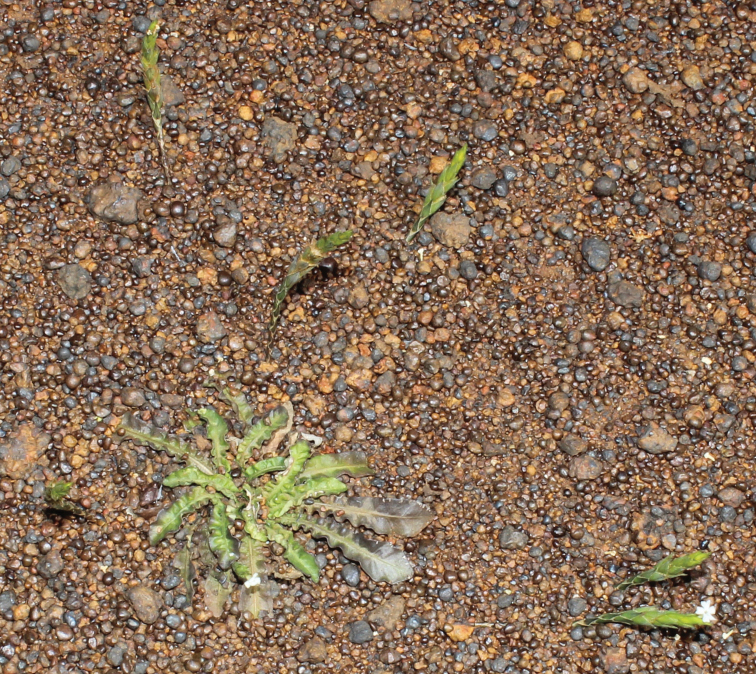
*Elytraria serpens*, a plant at the locus classicus, with six inflorescences (one of them with a flower, bottom right) emerging from the ground around it (photo R. A. Pérez Obregón).

“Mocarrero” soils cover large areas in the Cuba lowlands. The particular locality to which we refer is, moreover, characterised by the presence of a dense and homogeneous surface layer of ferralitic glomeruli, conditioning a very open vegetation interrupted by sterile gravel patches (Fig. 1). In Cuba, ± globular concretions of this type are found in various types of soil, including “mocarrero”. In pedology, they are known as “perdigón” or pellets (Bennett and Allison 1928: 4–6); they are thought of as matching the “mocarrero” ironstone’s mineral composition (Bennett and Allison 1928: 78–80) and they are considered as resulting from in situ concretion, not as alluvial deposits.

It would be interesting, although exceeding the purpose of this paper, to study the microclimatic properties of the pellet surface layer in our locality. We surmise that the thermal and hydric conditions inside and below the gravel layer will be found to differ markedly from those at the soil surface and in the overlying air.

No exhaustive inventory of the flora present in that locality was made, but a good sample of the flora was collected, including the following taxa (listed alphabetically with, in parentheses, the collecting number of the voucher specimens [kept in the herbaria PAL-Gr, B, HAJB and ULV]): *Angelonia pilosella* J. Kickx f. (29683), *Aniseia martinicensis* (Jacq.) Choisy (29684), *Byrsonima crassifolia* (L.) Kunth (29696), Caesalpinia pinnata subsp. oblongifolia (Urb.) A. Barreto & Beyra (29681), *Cuphea parsonsia* (L.) R. Br. (29693), *Evolvulus minimus* Ooststr. (29685), *Metastelma cubense* Decne. (29686), *Rauvolfia cubana* A. DC. (29689), *Paspalum edmondii* (29695), *Phyla stoechadifolia* (L.) Small (29692), *Pisonia rotundata* Griseb. (29691), *Sideroxylon salicifolium* (L.) Lam. (29694), *Stachytarpheta angustifolia* (Mill.) Vahl (29682), *Tabebuia lepidota* (Kunth) Britton (29690) and *Viguiera dentata* (Cav.) Spreng. (29688). According to Borhidi’s (1991: 613) classification, that plant community would seem to belong to the class *Curatello*-*Byrsonimetea* Borhidi and perhaps to the order *Byrsonimo*-*Andropogonetalia teneris* Bal.-Tul.: low-grass savannahs with scattered palms, on seasonally flooded “mocarrero” soil.

**Figure 2.**
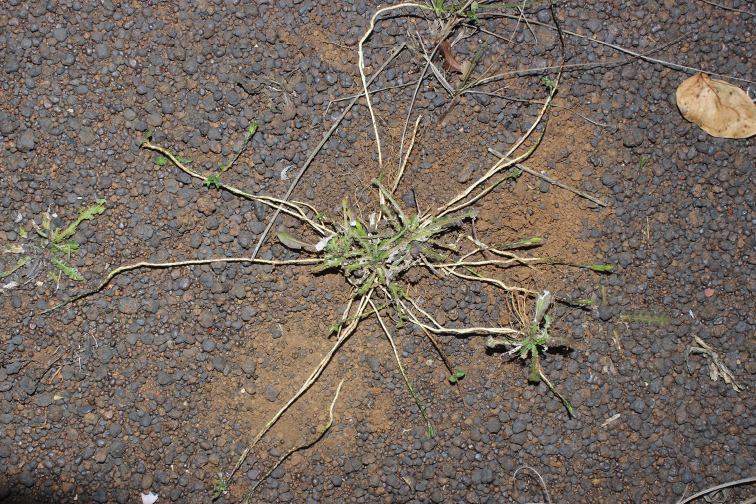
*Elytraria serpens*, a plant at the locus classicus with a dozen unearthed stoloniform peduncles, showing several subapical buds (rosettes in formation) and one fully developed daughter rosette, already rooted (photo R. A. Pérez Obregón).

Scattered on the gravel patches of that locality, we noticed a very curious plant, a species of the genus *Elytraria* Michx. (*Acanthaceae*) that did not match any of the known Cuban representatives of this genus: stemless perennial herbs, with all leaves flat on the ground, forming a basal rosette. The inflorescences, solitary or paired spikes, emerge from the soil at a distance from their rosette and are connected with it by a slender axis (the peduncle) winding below or inside the top layer of gravel. These peduncles, which mimic underground stolons, are pale, yellow or yellowish-brown in colour and covered with dense scales or sterile bracts. In the flowering season, they are devoid of adventitious roots, but at their apex, below the inflorescence(s), they frequently produce buds that develop into small foliar rosettes, destined to take root and give rise to new plants in the subsequent rainy season (Fig. 2). The spikes, surrounded by densely imbricate bracts, produce few tiny, white, unspectacular flowers that open one at a time. It is hard to believe that they are apt to attract pollinating flying insects; which is why it is likely that pollination, when performed, is the work of small terrestrial animals, for example, ants. It is obvious that reproduction is mostly vegetative, a hypothesis that explains the scarcity of well-developed fruits and the low efficiency of the seed dispersal mechanism.

## Taxonomy

### Elytraria serpens

Taxon classificationPlantaeLamialesAcanthaceae

Greuter & R. Rankin sp. nov.

CC971C37-BBB2-5255-8FBE-CB8BE69AB7B1

urn:lsid:ipni.org:names:77217118-1

#### Type.

**Figure 3.**
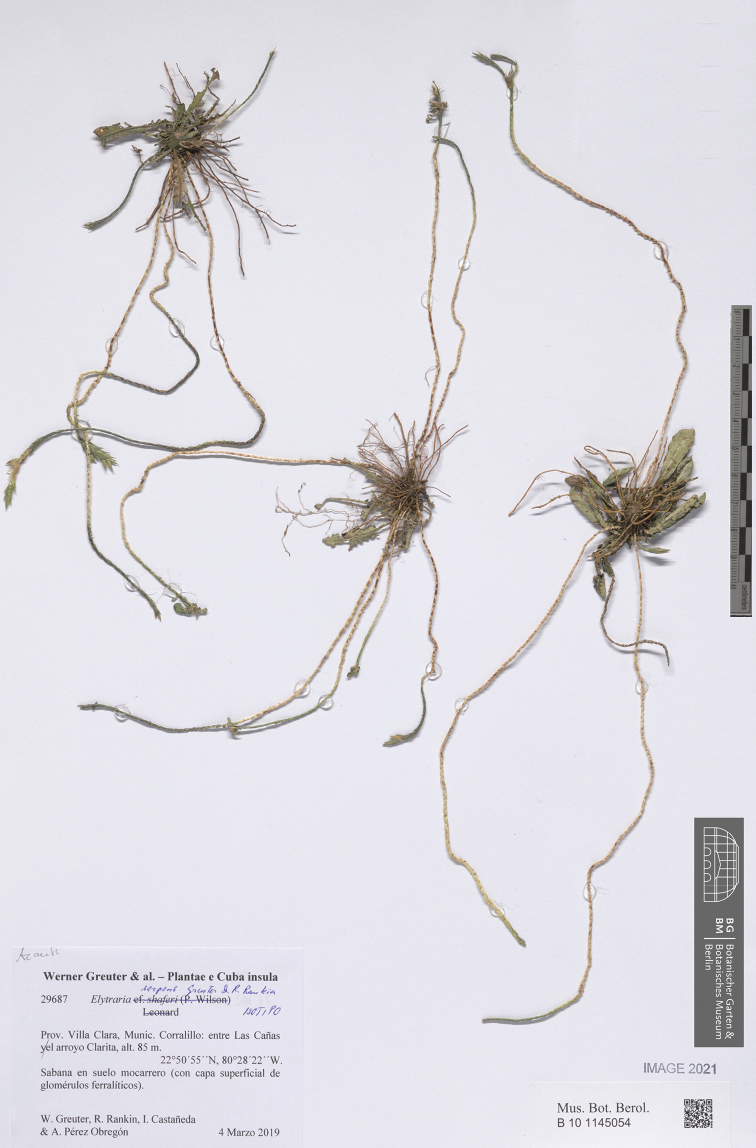
Isotype of *Elytraria serpens* (B).

Cuba central, Prov. Villa Clara, “Municipio Corralillo: entre Las Cañas y el arroyo Clarita, alt. 85 m, 22°50'55"N, 80°28'22"W. Sabana en suelo mocarrero (con capa superficial de glomérulos ferralíticos)”, 4-III-2019, *Greuter 29687*, *R. Rankin*, *I. Castañeda & A. Pérez Obregón* (holotypus PAL-Gr, isotypi: B #101145054 [Fig. 3], HAJB, JE, ULV).

Planta perennis herbacea acaulis,foliis omnibus basalibus in rosulam humo accumbentem congestis. Folia anguste spatulata, 2–4 cm longa et 0.6–0.8 mm lata, subplana vel saepius transverse undulata, glabra vel praecipue in latere abaxiali ad costam ± villosa, petiolo brevi (2–3 mm) pallide brunneo-villoso. *Pedunculi* graciles, 3–12 ex axillis rosulae basalis orientes, unus alterve brevis arcuate adscendens folia vix superans, praecipui autem tortuosi, stolonorum modo longe (20 cm vel ultra) subterranee repentes, omnes dense bracteis sterilibus squamiformibus subimbricatis obsiti. *Bracteae steriles* sessiles, amplexicaules, subterraneae, extus glabrae, margine antrorsum ciliolatae, intus apicem versus minutissime glanduloso-papillosae; inferiores (subterraneae) ovato-triangulares, subacutae, stramineae, minimae (1–2 mm longae), superiores (aereae) gradatim majors, 2–3 mm longae, acuminatae, virentes. Ad apicem pedunculorum nonnullorum *gemmae* jam florendi tempore foliiferae sed nondum radicantes conspiciuntur. *Inflorescentiae* spiciformes, densae, 1–2 cm longae, bracteis imbricatis indutae, ad apicem pedunculorum singulae vel binae ternaeve congestae. *Bracteae floriferae* bracteis sterilibus superioribus non dissimiles sed majores, virides, ca. 6 mm longae et 2.5 mm latae, ovato-triangulares, acuminatae et breviter aristatae, infra glabrae sed apicem versus sub lente retrorso-pubescentes, margine cuncto antrorse ciliatae, intus minute glanduloso-papillosae. *Flores* pauci, singulatim florentes. *Calyx* ca. 5 mm longus, bracteolis binis suffultus. *Bracteolae* bracteis conformes sed minores et angustiores, ca. 4 mm longae et 0.7 mm latae. *Sepala* 4 (sed sepalum abaxiale binerve et apice bidentatum), bracteolis majora, ca. 5 mm longa et 1.2 mm lata, extus glabra, margine apicem versus fimbriato-ciliata, intus minute antrorso-puberula. *Corolla* parva (ca. 3 mm longa), hypocrateriformis, albida, tubo subrecto, inconspicue sigmoideo, ca. 2.5 mm longo et 0.6 mm crasso, limbo subregulari, 1.5 mm diámetro, lobis expansis truncato-retusis. *Capsulae* (paucae perfectae) lineari-obpyriformes, ca. 4 mm longae et 1.2 mm latae, bivalvatae, valvis post dehiscentiam basi connatis apice arcuatim divergentibus. *Semina* (submatura?) ca. 12, quae dehiscencia peracta in capsula inclusa manent, pallide brunnea, glabra (etiamsi in acua inmersa), subanguloso-ellipsoidea, ca. 0.6 mm longa et 0.4 mm lata, sub lente rugulosa.

Amongst Cuban *Acanthaceae*, the two genera *Elytraria* and *Stenandrium* Nees stand out, being small stemless herbs with basal leaves forming a rosette and flowers in terminal spikes borne on scapiform peduncles that emerge directly from the basal rosette. According to Alain (1957) and other authors, these genera are best distinguished by the number of fertile stamens, 2 in the first and 4 in the second; but this character is not easily observed even when flowers are present. More obvious is the difference in the scapes or peduncles, which are naked in *Stenandrium*, but densely beset with small scaly bracts in *Elytraria*. In addition, the inflorescences of the latter genus are compact, surrounded by densely imbricate bracts, whereas, in *Stenandrium*, at least the lower flowers of each spike are distant from each other and their bracts do not overlap. Both genera have a major centre of diversity in the Caribbean. According to Greuter and Rankin Rodríguez (2017), seven taxa (species and subspecies) of *Elytraria* were known from Cuba, all endemic and 11 taxa of *Stenandrium*, nine endemic. Worldwide (PoWo 2021), 22 taxa are accepted in *Elytraria*: the seven Cuban ones, plus one from Hispaniola, eight from continental America, four from the African continent (one of them also grows in eastern India) and two from Madagascar. The genus *Stenandrium* is larger (65 taxa) and less homogeneous, as it includes several species with elongated stems and opposite leaves; it presents a very similar distribution and diversification pattern: 15 taxa from the Caribbean islands, 11 of them present and nine endemic in Cuba, more than 31 species from the American continent (22 from South America); and 19 African species, 10 of them endemic to Madagascar (PoWo 2021).

Most of the seven Cuban endemics of *Elytraria* have restricted ranges and five of the six species are threatened; one of them (*E. filicaulis*) is considered as Critically Endangered (González-Torres et al. 2016). Borhidi and Muñiz (1978), who proposed a determination key for the six taxa known at that time, distinguish species with papery leaves and 5-merous calyx from the others, with membranous leaves and 4-merous calyx. However, our data and the descriptions of other authors (Leonard 1934; Dietrich 1982) suggest that the calyx always consists of four free sepals, but with the abaxial one slightly wider, 2-nerved and apically bidentate, that takes the place of two concrescent sepals. According to label data and protologue indications, at least four of the Cuban taxa of *Elytraria* grow on ophiolitic substrates and can be considered serpentinophytes: *E. cubana*, *E. filicaulis*, E. planifolia subsp. planifolia and E. planifolia subsp. acunae. *E. bissei* and *E. spathulifolia* are considered calcicolous, while the habitat of *E. shaferi*, according to the collector, is an arid cliff-face in a serpentine area of Holguín (Sierra de Nipe, Woodfred mines).

In its vegetative features, *Elytraria serpens* is very similar to *E. bissei* of limestone areas of southern Guantánamo (Abra de Mariana), which, however, has leaves hairy on both sides and subsessile spikes not exceeding the basal rosette; and it is akin to *E. shaferi*, with which it shares the pubescence of the outer face of the flower bracts. The species most closely related to ours is *E. cubana*, for which the collector, on the label of the type specimen (*Shafer 2948*, NY), noted: “Lvs. flat on ground, among rocks in red soil, stony hillsides”. In this species, with leaves of similar dimensions and shape to ours, the peduncles are decumbent and flexuous, but much shorter than in *E. serpens*, never subterranean, and the flower bracts, on the outside, are glabrous rather than pubescent.

In our opinion, *Elytraria serpens* evolved from plants similar to *E. cubana* by adapting itself to the particular edaphic conditions of its habitat. It takes advantage of the loose granular structure of the gravel layer that enables it to push its developing peduncles through it, hiding them underground, sheltered from the extreme drought and radiation at the surface, to produce apical buds at an appreciable distance from its origin, thus ensuring its vegetative spread. The specimens at hand suggest that fruit set is poor, perhaps due to inadequate pollination and that the explosive capsule dehiscence, normally ensuring seed dispersal in this family (Greuter and Rankin Rodríguez 2010), is not fully functional.

## Supplementary Material

XML Treatment for Elytraria serpens

## References

[B1] Alain (1957) Flora de Cuba, 4. Dicotiledóneas: *Melastomataceae* a *Plantaginaceae*. Contribuciones Ocasionales del Museo de Historia Natural del Colegio “De La Salle” 16.

[B2] Bennett HH Allison RV (1928) The Soils of Cuba. Washington.

[B3] Borhidi A (1991) Phytogeography and Vegetation Ecology of Cuba. Budapest.

[B4] Borhidi A Muñiz O (1978) Notas sobre Acantáceas cubanas I. *Oplonia* y *Elytraria*. Acta Biologica Academiae Scientiarum Hungaricae 23: 303–317.

[B5] Dietrich H (1982) *Acanthaceae* cubanae novae I. *Elytraria bissei* H. Dietrich, spec. nov. Revista del Jardín Botánico Nacional, Universidad de La Habana 3(2): 39–51.

[B6] González-Torres LR Palmarola A González-Oliva L Bécquer ER Testé E Barrios D (2016) Lista roja de la flora de Cuba. Bissea 10, Núm Espec: 1–1.

[B7] Greuter W Rankin Rodríguez R (2010) Notes on some endemic Cuban species of Ruelliinae (Acanthaceae), on their seeds, pollen morphology and hygroscopic features. Willdenowia 40(2): 285–304. 10.3372/wi.40.40210

[B8] Greuter W Rankin Rodriguez R (2017) Plantas vasculares de Cuba: inventario preliminar. Segunda edición, actualizada, de Espermatófitos de Cuba con inclusión de los Pteridófitos. Vascular plants of Cuba: a preliminary checklist. Second updated edition of The *Spermatophyta* of Cuba with *Pteridophyta* added. Berlin & La Habana. 10.3372/cubalist.2017.1

[B9] Leonard EC (1934) The American species of *Elytraria*. Journal of the Washington Academy of Sciences 24: 443–447.

[B10] PoWo (2021) PoWo, Plants of the World online. Kewscience. http://www.plantsoftheworldonline.org/

